# Operational nurse managers’ perceptions on the competence of community service nurses in public settings in the Western Cape

**DOI:** 10.4102/curationis.v44i1.2174

**Published:** 2021-03-31

**Authors:** Vatiswa Makie, Karien Jooste, Tendani B. Mabuda, Theresa Bock, Guinevere M. Lourens, Martha van As, Jennifer Chipps

**Affiliations:** 1 Western Cape College of Nursing, Cape Town, South Africa; 2 Department of Nursing Science, Cape Peninsula University of Technology, Cape Town, South Africa; 3 Department of Nursing and Midwifery, Stellenbosch University, Cape Town, South Africa; 4 School of Nursing, University of the Western Cape, Cape Town, South Africa

**Keywords:** competencies, community service nurse, professional nurses, public healthcare, clinical patient care

## Abstract

**Background:**

Community service nurses placed in the Western Cape Government public health facilities render essential healthcare to underserved populations. Anecdotal evidence from operational nurse managers indicated concerns that community service nurses may lack competence in basic required nursing competencies.

**Objectives:**

To investigate operational nurse managers’ perceptions of the competence of community service nurses in public health facilities in the Western Cape.

**Method:**

A quantitative survey was conducted with an all-inclusive sample of 297 operational nurse managers in the Western Cape. A self-administered questionnaire with 65 questions with a 4-point rating scale was used to rate perceived competence of community service nurses across the South African Nursing Council (SANC) competencies. Descriptive and inferential statistics were calculated per competency domain.

**Results:**

The survey (response rate: 59%) showed that the operational nurse managers perceived the community service nurses to be competent in the clinical patient care domain and mostly either developing proficiency or proficient in the SANC competencies of legal framework and ethical practice, interprofessional relationships, leadership, quality management and management competency domains.

**Conclusion:**

Community service nurses were found to be competent in the clinical patient care, possibly because of the integration of theory and practice focus of work-integrated learning in the programme. Education and practice supportive strategies for community service nurses should be developed to support the successful transition from students to community service nurses, especially around the development of research and critical thinking skills.

## Introduction

Competent nurses can make an impact on quality and cost-effective healthcare in South Africa (Maphumulo & Bhengu 2019). The National Nursing Strategy (2012/13–20/16/17) states that South Africa’s healthcare system, which is predominantly nurse-based, requires nurses inclusive of community service nurses to have the competence to address the country’s burden of disease to contribute to the healthcare needs of the community. Nurses play a fundamental role in the achievement of the Western Cape Departmental Healthcare 2030 priority focus areas which are to provide a comprehensive quality care service from primary healthcare to highly specialised services (Western Cape Government Healthcare 2017).

Nursing competency is commonly viewed as a complex integration of knowledge, including professional judgement, clinical skills, values and attitudes, which is applied in each situation and adapted to different circumstances (Fukada 2018). At the time of the study, the South African Nursing Council (SANC) prescribed the requirements to be competent when completing a 4-year integrated, comprehensive programme, leading to the registration as a nurse (general, psychiatric and community) and midwife (Regulation R425 of 22 February 1985, as amended [SANC 1985]). The integrated 4-year diploma or degree qualification which is provided at a university or college was implemented in 1984 in line with the nurse-based primary healthcare approach of South Africa. On completion of the qualification, new graduates intending to register for the first time to practise a profession in a prescribed category must perform remunerated community service for a period of 1 year at a public health facility (SANC 2007). Only designated public health establishments can be utilised to perform community service (SANC 2007).

The reason for the implementation of compulsory community service is the Department of Health’s intention to recruit and retain healthcare professionals within South African public sector and to improve access to quality healthcare to all South Africans, more especially in previously under-served areas (Mchunu 2006; South African Department of Health 2006). In 2008, the first community service nurse cadre started with community service in the Western Cape.

Nurses are expected to take professional responsibilities for continuously providing direct care, protecting individual lives and supporting activities of daily living (Fukada 2018). When nursing students graduate and become employees of the healthcare industry, they are recognised as key participants in provision of healthcare as competent caregivers in the delivery of safe patient care. Incompetent graduate nurses will lead to inability to meet the needs of caring for the complex patient population (Keshk & Mersal 2017).

A study on competence of new graduate nurses identified several competencies that are essential for new nurse graduates, including communication, leadership, organisation and critical thinking (Theisen & Sandau 2013). These competencies are linked with clinical patient care, leadership and management. Similarly, the most important skills for new graduate nurses were identified as administering and monitoring therapeutic interventions and regimens, monitoring and ensuring quality of healthcare practices and organisational and work-role competencies (Keshk & Mersal 2017). The competencies for community service nurses on entry into employment are set out by SANC in the following competency domains: Professional Ethical Practice; (Legal, Ethical and Accountability) Clinical Practice; Care Provision and Care Management; and Quality Management (SANC 2004). International studies conducted on competencies of graduate nurses highlight similar domains.

Various studies in South Africa have been conducted on the perceptions and experiences of community service nurses (Govender, Brysiewicz & Bhengu 2015; Ndaba & Nkosi 2015; Roziers, Kyriakos & Ramugondo 2014; Shezi 2014; Zaayman 2016). Studies showed that community service nurses experience reality shock with stress, fear and uncertainty, along with feelings of being overwhelmed, role confusion, poor staff attitudes towards them, lack of motivation, less confidence and difficulties in applying new knowledge (Ndaba & Nkosi 2015; Roziers et al. 2014; Shezi 2014). Nkoane (2015) and Zaayman (2016) pointed out that these findings suggest a crisis across required competencies compounded by inadequate undergraduate programme preparation, lack of orientation and professional support during community service.

### Problem statement

Annually, there are about 376 community service nurses placed in the Western Cape Government public health facilities gazetted for community service (WCDOH 2019). Community service nurses are often placed in areas where they have to work independently within the first year after qualifying as a graduate, without being supervised and supported in the public health facility with an expectation to be competent across all the core SANC competencies (Ndaba & Nkosi 2015; Roziers et al. 2014; Shezi 2014). Anecdotal evidence from operational nurse managers during provincial meetings had indicated perceptions that community service nurses may lack competence in the basic required nursing competencies. Few studies have been conducted on the perceptions of operational nurse managers on the competence of these community service nurses across a range of nursing competencies.

### Aim

The aim of the study was to investigate operational nurse managers’ perceptions of the competence of community service nurses placed in public healthcare facilities in the Western Cape.

## Research methods and design

### Design

A quantitative descriptive survey using a self-administered questionnaire was used to conduct the study. A survey was the most appropriate method to investigate the aim across a wide number of operational managers in different facilities.

### Setting

In each of the district or sub-district’s substructure, an average of 1–33 community service nurses are placed annually. The setting for the study was all the 69 gazetted public hospital and primary healthcare facilities in both metro and rural healthcare services in the Western Cape where community service nurses are allocated. This study was conducted with the nursing operational managers in these facilities.

### Study population and sampling strategy

The accessible population of the study was 297 operational nurse managers working in facilities gazetted for community service in both rural and metro healthcare facilities in the Western Cape. All-inclusive sampling was used and all operational nurse managers working in facilities where community services nurses were placed were requested to participate in the study.

### Instrument

Data were collected using a self-administered questionnaire which was developed based on the literature and the SANC framework of professional nurses’ competencies in South Africa (SANC 2004). The questionnaire had three sections: Section 1: Demographic data; Section 2: Competence ratings and Section 3: Recommendations for improvements. In Section 2, the competence ratings were provided using a 4-point Likert rating scale which was developed to measure perceived competence across four ratings: does not demonstrate (0), developing competence (1), proficient (2) and advanced (3). These ratings were defined as: *Advanced competence indicates that the nurse demonstrated a broad and deep understanding and skills, with substantial experience in this area as a role model. A proficient competence referred to the nurse having adequate understanding and experience to generalise basic principles to effectively function in both predictable and new situations. Developing competence referred to the nurse having a general understanding of key principles but limited or no applied experience. Incompetent referred to a nurse who did not demonstrate competency at the expected level, even with available assistance or direction from others*. An explanation of the rating definitions was provided on the questionnaire.

Face validity of the instrument was established through key stakeholders’ workshops to develop the questionnaire and to provide subjective confirmation that the instruments are measuring what it purports to measure. Content validity, to ensure that all facets of community service nurses’ competencies were included, was established within the SANC Competency Framework (SANC 2004). Reliability was measured by calculating a scale reliability score for internal consistency using Cronbach’s alpha (*α* = 0.995). A pre-test of the instrument was conducted with 10 operational nurse managers in the metro district and no changes were made. These respondents were therefore also included in the main study.

### Data collection

Data collection started in late 2017 and completed by March 2018. An email directive was sent from the Office of the Director of Nursing Services to all operational nurse managers inviting them to complete the survey. In addition, one of the researchers distributed the questionnaires to the service providers and followed this up during a meeting of operational nurse managers where the value of this was explained to attendees prior to inviting them to complete the questionnaire. No identifying information was collected from respondents. They were requested to complete the questionnaire on the same day. Questionnaires did not take longer than 30 min to complete and were returned in a closed envelope to the researcher. Respondents completed the questionnaire in English.

#### Data analysis

The data were analysed using the SPSS version 25 software program. Demographic data were described using descriptive statistics. All competencies were reported by the extent (level) to which a respondent was competent using number of responses (*n*), mean and standard deviation (s.d.) and (95% confidence intervals [CIs]) for each competency and one sample *T*-test with the test value set at developing (rated 1) was conducted, with significance set at *p* < 0.05.

### Ethical considerations

Ethics approval (BM17/7/7) was received from the University of Western Cape Ethics Committee. Approval to conduct the study was sought from the Provincial Government of the Western Cape and the Department of Health granted permission to enter public facilities in the Western Cape. Participation in the study was voluntary and anonymity was assured. The respondents were informed of their right to withdraw from the process at any stage of the project, without prejudice. Respondents were informed that all data would be treated as confidential and written consent was provided.

## Results

A total of 167 operational nurse managers completed questionnaires out of a total of 297 (56% response rate). One questionnaire was discarded as none of the competencies were rated, leaving a total of 166 responses. Most of the respondents were females (151, 91% female; 5, 9% male) with an average age of 49.5 years (s.d. = 7.3 years) with the age ranging from 32 to 64 years old. Nearly three-quarters of the respondents reported their highest qualification as a diploma in nursing (117, 70.5%) with 45 respondents (27.1%) reporting a degree in nursing. The average time the respondents had been employed in nursing was 23.8 years (s.d. = 8.2 years), ranging from 2 to 41 years. Most of the respondents were from primary healthcare services (including district hospitals) (88, 41.5%) followed by respondents from academic hospitals (56, 36%).

### Overall perceived competency

The perceptions of competence were measured using a 4-point scale rating on the individual competencies in the domains of legal framework and ethical practice, clinical patient care, interprofessional relationships, leadership, quality management and management.

**FIGURE 1:**
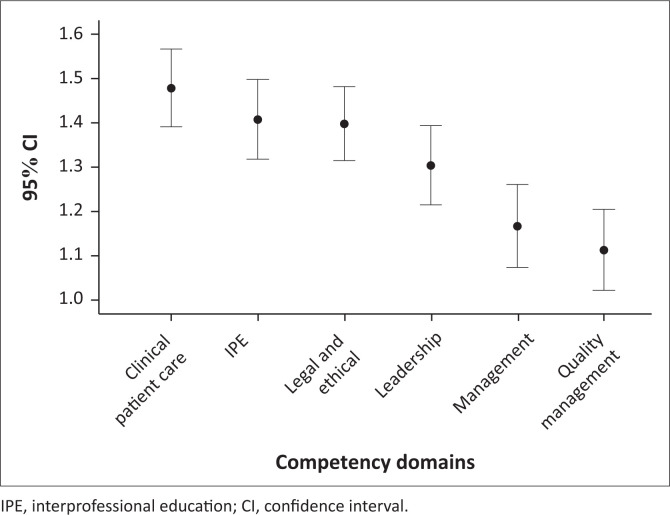
Competence ratings of the competency domains.

Clinical patient care was the highest rated domain (*m* = 1.48 [95% CI: 1.39–1.56]), followed by interprofessional (*m* = 1.41 [95% CI: 1.32–1.50]) and legal and ethical domains (*m* = 1.40 [95% CI: 1.32–1.48]) (Figure 1). The lowest rated domain was quality management (*m* = 1.11 [95% CI: 1.02–1.20]) (Figure 1). When comparing the perceptions of the respondents on competencies of the community service nurses across the six competency domains, clinical patient care was rated significantly higher than leadership, management and quality management and quality management was rated significantly lower than all other domains (Figure 1). The highest mean rating value was for *monitoring vital signs* (2.2, s.d. = 0.7) and the lowest mean rating was for *using written feedback and reporting system* (1.2, s.d. = 0.7).

### Clinical patient care

Clinical patient care was the highest rated competency domain (1.48/3, s.d. = 0.57). All the mean ratings for the competencies were significantly higher than 1 (*developing competence*) with the highest mean rating for *monitoring vital signs* (2.2, s.d. = 0.7). The lowest mean rating value was for *developing accurate nursing care plans based on individual needs* (1.2, s.d. = 0.8) (Table 1).

**TABLE 1:** Competency ratings for clinical practice domain.

Competencies	Mean	s.d.	Does not demonstrate		Developing		Proficient		Advanced	
			*n*	%	*n*	%	*n*	%	*n*	%
General observations of pulse, temperature, blood pressure and urine test (*n* = 158)	1.9	0.7	5	3.2	30	19	98	62	25	15.8
Haemoglucose test, haemoglobin (*n* = 163)	1.9	0.7	5	3.1	36	22.1	97	59.5	25	15.3
Management of patient receiving nebuliser therapy (*n* = 150)	1.8	0.7	5	3.3	41	27.3	88	58.7	16	10.7
Management of patient receiving oxygen therapy (*n* = 144)	1.8	0.7	3	2.1	41	28.5	85	59	15	10.4
Check and keep record of medication appropriately (*n* = 157)	1.7	0.7	7	4.5	42	26.8	94	59.9	14	8.9
Administration of medication according to the correct principles (*n* = 164)	1.7	0.7	7	4.3	50	30.5	94	57.3	13	7.9
Undertaking the correct calculations to administer dosages (*n* = 161)	1.7	0.6	4	2.5	52	32.3	96	59.6	9	5.6
Maintenance of fluid balance (*n* = 152)	1.6	0.7	12	7.9	50	32.9	81	53.3	9	5.9
Monitoring a patient receiving intravenous therapy (*n* = 141)	1.6	0.7	7	5.0	53	37.6	72	51.1	9	6.4
Document interventions and progress of client status to facilitate continuity of care (*n* = 162)	1.6	0.7	8	4.9	65	40.1	80	49.4	9	5.6
Observe the patient pre- and post-operative (*n* = 132)	1.6	0.7	5	3.8	51	38.6	68	51.5	8	6.1
Supervision of a patient with blood transfusion (*n* = 134)	1.5	0.7	10	7.5	55	41.0	60	44.8	9	6.7
Provide emotional support to patients and families (*n* = 162)	1.5	0.7	12	7.4	70	43.2	70	43.2	10	6.2
Ensure the safe administration of therapeutic substances (*n* = 152)	1.5	0.7	6	3.9	68	44.7	69	45.4	9	5.9
Conducts the necessary physical and mental assessments (*n* = 165)	1.4	0.8	21	12.7	72	43.6	61	37	11	6.7
Facilitate health promotion and education (*n* = 162)	1.4	0.8	20	12.3	70	43.2	64	39.5	8	4.9
Collect and analyse data of the patient by taking a history (*n* = 165)	1.4	0.7	13	7.9	78	47.3	64	38.8	10	6.1
Monitor progress and outcome of interventions on the physical, psychological and psychosocial well-being of individuals (*n* = 160)	1.4	0.7	12	7.5	81	50.6	61	38.1	6	3.8
Assess the patient to formulate an accurate nursing care diagnosis (*n* = 162)	1.3	0.8	22	13.6	72	44.4	60	37.0	8	4.9
Neurological assessment (*n* = 124)	1.3	0.8	14	11.3	61	49.2	41	33.1	8	6.5
Document the plan of care to facilitate communication with other healthcare team members for continuity of care (*n* = 158)	1.3	0.7	19	12.0	80	50.6	52	32.9	7	4.4
Performing basic cardiopulmonary resuscitation (*n* = 143)	1.3	0.7	17	11.9	78	54.5	42	29.4	6	4.2
Develop accurate nursing care plans based on individual needs (*n* = 160)	1.2	0.8	27	16.9	81	50.6	44	27.5	8	5.0

s.d., standard deviation.

*Developing accurate nursing care plans* received the highest perception rating of not being able to demonstrate this skill (16.9% of respondents). When considering the item on demonstrating skills in *medication*, namely, *check and keep record of medication appropriately, administration of medication according to correct principles* and undertaking the correct calculations to administer dosages, only 4.5%, 4.3% and 2.5% of the respondents rated community service nurse as not being able to demonstrate competencies in these fields (Table 1). The highest ratings were for *general observations* (1.9, s.d. = 0.7) and *haemoglucose test* (19, s.d. = 0.7) (Table 1).

### Interprofessional practice

This was the second highest rated competency domain after clinical patient care (1.4, s.d. = 0.59). All the mean ratings for the competencies were significantly higher than 1 (*developing competence*). The highest mean rating was for *establishing and maintaining constructive working relationships with nursing and other colleagues* (1.6, s.d. = 0.7), and the lowest mean rating *for using a system to ensure timeous and appropriate referral to members of the health team* (1.3, s.d. = 0.7) *and participating in decision-making pertaining to healthcare delivery* (1.3, s.d. = 0.7), which was also the highest rated interprofessional competency as ‘does not demonstrate’ by (21, 12.8%) of the respondents (Table 2).

**TABLE 2:** Competence ratings for interprofessional practice domain.

Competencies	Mean	s.d.	Does not demonstrate		Developing		Proficient		Advanced	
			*n*	%	*n*	%	*n*	%	*n*	%
Establishes and maintains constructive working relationships with nursing and other colleagues (*n* = 161)	1.6	0.7	6	3.7	62	38.5	83	51.6	10	6.2
Applies knowledge of effective inter-professional working practices (*n* = 162)	1.5	0.7	9	5.6	71	43.8	74	45.7	8	4.9
Participate in the doctors and the multidisciplinary team discussion/rounds and implement the instructions (*n* = 159)	1.4	0.7	18	11.3	67	42.1	68	42.8	6	3.8
Consult and collaborate within the multi-disciplinary health teams (*n* = 163)	1.4	0.7	18	11.0	71	43.6	68	41.7	6	3.7
Act as a role model for students in practice (*n* = 158)	1.4	0.7	13	8.2	73	46.2	65	41.1	7	4.4
Participate in decision-making pertaining to healthcare delivery (*n* = 164)	1.3	0.7	21	12.8	81	49.4	57	34.8	5	3.0
Use a system to ensure timeous and appropriate referral to members of the healthcare team (*n* = 157)	1.3	0.7	16	10.2	81	51.6	54	34.4	6	3.8

s.d., standard deviation.

### Legal framework and ethical practice

This was the third highest rated domain after clinical patient care and interprofessional relationships with a mean competence rating of 1.41/3 (s.d. = 0.59). All the mean ratings for the legal framework and ethical practice competencies were higher than 1 (*developing competence*), with the highest mean rating for *ensuring respect towards patients* (1.8, s.d. = 0.6) with only 4 (2.5%) of the respondents rating community service nurses as incompetent in this area. The lowest mean rating was for *implementing and monitoring occupational health and safety measures in accordance with the occupational health and safety legislation* (1.1, s.d. = 0.7) with 29 (18%) respondents also rating community service nurses as not demonstrating competence in this area (Table 3).

**TABLE 3:** Competence rating for legal framework and ethical practice.

Competencies	Mean	s.d.	Does not demonstrate		Developing		Proficient		Advanced	
			*n*	%	*n*	%	*n*	%	*n*	%
Ensures confidentiality and security of all information acquired in a professional capacity (*n* = 160)	1.8	0.6	4	2.5	52	32.5	93	58.1	11	6.9
Ensure the respect towards patients, for example, their right to privacy (*n* = 162)	1.7	0.6	3	1.9	42	25.9	99	61.1	18	11.1
Advocates for rights of patients on their behalf (*n* = 159)	1.4	0.7	16	10.1	64	40.3	72	45.3	7	4.4
Takes responsibility for own independent performances of tasks (*n* = 163)	1.4	0.7	15	9.2	77	47.2	67	41.1	4	2.5
Functions within legislative and common law affecting nursing care (*n* = 161)	1.4	0.6	7	4.3	90	55.9	61	37.9	3	1.9
Ensure security of assets in the unit (*n* = 163)	1.3	0.7	18	11.0	81	49.7	57	35.0	7	4.3
Identifies unsafe care practices and takes appropriate actions to combat them (*n* = 164)	1.2	0.7	24	14.6	88	53.7	45	27.4	7	4.3
Uses a formal system to verify the credentials (registration/enrolment status, knowledge, skills and experience) of the full-time, part-time and sessional/agency staff (*n* = 151)	1.2	0.7	24	15.9	77	51.0	44	29.1	6	4.0
Implements and monitors occupational health and safety measures in accordance with the occupational health and safety legislation (*n* = 161)	1.1	0.7	29	18.0	87	54.0	40	24.8	5	3.1

s.d., standard deviation.

### Leadership

The Leadership domain was the third-lowest rated domain after management and quality management (1.3, s.d. = 0.58). The mean ratings of all the competencies in the domain, except one, were significantly higher than 1 (*developing competence*) with the highest rating for *developing an atmosphere of teamwork and cooperation* (1.5, s.d. = 0.7), and the lowest rating *for influencing staff to focus on outcomes of quality health* (1.1, s.d. = 0.7). *Influencing staff to focus on outcomes of quality health Influence staff to focus on the outcomes of quality health* (1.1, s.d. = 0.7) also received the highest proportion of respondents rating community service nurses as not being able to demonstrate this skill (29, 17.7% of respondents) (Table 4).

**TABLE 4:** Competence rating for leadership.

Competencies	Mean	s.d.	Does not demonstrate		Developing		Proficient		Advanced	
			*n*	%	*n*	%	*n*	%	*n*	%
Promotes trust and open exchange of ideas (*n* = 165)	1.5	0.7	15	9.1	69	41.8	72	43.6	9	5.5
Set an example in continuous professional development of the self (*n* = 166)	1.3	0.7	21	12.7	83	50.0	56	33.7	6	3.6
Stay accountable for delegated responsibility based on abilities of staff (*n* = 161)	1.3	0.7	16	9.9	85	52.8	53	32.9	7	4.3
Have the knowledge to guide staff to obtain the goals of the unit (*n* = 162)	1.2	0.7	27	16.7	89	54.9	40	24.7	6	3.7
Promote interactive decision-making and problem-solving amongst staff (*n* = 164)	1.2	0.7	27	16.5	86	52.4	47	28.7	4	2.4
Resolves conflict positively (*n* = 162)	1.2	0.7	24	14.8	93	57.4	38	23.5	7	4.3
Use her/his authority to instil respect of staff for the quality of life (*n* = 165)	1.2	0.7	22	13.3	89	53.9	49	29.7	5	3.0
Have a vision in coordinating activities in the multidisciplinary team (*n* = 165)	1.2	0.7	18	10.9	95	57.6	47	28.5	5	3.0
Influence staff to focus on outcomes of quality health (*n* = 164)	1.1	0.7	29	17.7	92	56.1	40	24.4	3	1.8

s.d., standard deviation.

### Management

The mean competency rating for the domain of management was (1.16, s.d. = 0.60) and this domain was the second lowest domain. All the mean ratings for the competencies were significantly higher than 1 (*developing*) with the highest mean rating for *planning daily activities within the policies of the service* (1.3, s.d. = 0.7), and the lowest mean rating for *using a formal procedure for determining the needs* (*stock, supplies and equipment*) *of the unit* (1.1, s.d. = 0.7). *Using a formal procedure for determining the needs (stock, supplies and equipment*) also was rated by 37 (23%) of the respondents as ‘does not demonstrate’ (Table 5).

**TABLE 5:** Competence rating for management.

Competencies	Mean	s.d.	Does not demonstrate		Developing		Proficient		Advanced	
			*n*	%	*n*	%	*n*	%	*n*	%
Use a formal procedure for determining the needs (stocks, supplies and equipment) of the unit (*n* = 161)	1.3	0.7	37	23	80	49.7	41	25.5	3	1.9
Use a written feedback and reporting system (*n* = 161)	1.2	0.7	26	16.1	74	46	58	36.0	3	1.9
Demonstrate an understanding of basic ward and financial control and procedures (*n* = 161)	1.1	0.7	30	18.6	91	56.5	39	24.2	1	0.6
Planning daily activities within the policies of the service (*n* = 165)	1.1	0.7	17	10.3	88	53.3	57	34.5	3	1.8

s.d., standard deviation.

### Quality management

Quality management was the lowest rated domain with a mean competence rating of (1.1, s.d. = 0.59). The mean ratings of seven of the competencies were significantly higher than 1 (*developing competence*) with the highest rating for *ensuring that the Batho Pele principles are implemented* (1.4, s.d. = 0.7), and the lowest rating *for nursing staff demonstrate insight into latest research findings and recommendations applicable to the specialised nursing/midwifery care* (1.9, s.d. = 0.7). This also was the lowest rating of all the competencies in the questionnaire. *Nursing staff demonstrating insight into the latest research finding and recommendations applicable to specialised midwifery/nursing care* also received the highest proportion of respondents rating community service nurses as not being able to demonstrate this skill (31.6% of respondents) (Table 6).

**TABLE 6:** Competence rating for quality management.

Competencies	Mean	s.d.	Does not demonstrate		Developing		Proficient		Advanced	
			*n*	%	*n*	%	*n*	%	*n*	%
Ensure that the Batho Pele principles are implemented (*n* = 164)	1.4	0.7	16	9.8	70	42.7	71	43.3	7	4.3
Ensure that duties are delegate according to scope of practice (*n* = 162)	1.3	0.8	23	14.2	65	40.1	69	42.6	5	3.1
Ensure that the incident reporting policy is implemented (*n* = 165)	1.3	0.7	23	13.9	80	48.5	57	34.5	5	3.0
Monitor the work procedures of staff members (*n* = 161)	1.2	0.7	27	16.8	83	51.6	48	29.8	3	1.9
Implements procedures that maintain effective infection control (*n* = 162)	1.2	0.7	21	13.0	87	53.7	49	30.2	5	3.1
Fulfil a continuous supervisory role to combat risks (*n* = 158)	1.1	0.7	32	20.3	86	54.4	37	23.4	3	1.9
Have an orientation programme for new staff members (*n* = 153)	1	0.8	40	26.1	72	47.1	37	24.2	4	2.6
Set standards to be followed to ensure correct procedures (*n* = 161)	1	0.7	40	24.8	90	55.9	26	16.1	5	3.1
Demonstrate knowledge of risk management, for example, disaster plan (*n* = 164)	1	0.7	35	21.3	89	54.3	38	23.2	2	1.2
Undertake an audit process to take corrective measures in the unit/facility (*n* = 154)	0.9	0.8	48	31.2	75	48.7	28	18.2	3	1.9
Nursing staff demonstrate insight into latest research findings and recommendations applicable to the specialised nursing/midwifery care (*n* = 155)	0.9	0.7	49	31.6	77	49.7	27	17.4	2	1.3
Update the unit circular file and guidelines for use at orientation of staff (*n* = 159)	0.9	0.7	46	28.9	88	55.3	21	13.2	4	2.5

s.d., standard deviation.

## Discussion

This study is the first study to examine the perceptions of operational nurse managers on the perceived competence of community service nurses. Overall, for community service nurses, most of the competencies were perceived to be between the competence ratings of *developing competency* and *being proficient.* Only 3.1% of the respondents rated community service nurses not perceived to be competent in glucose and haemoglobin testing. In contrast, 31.6% of the respondents rated community service nurses as not demonstrating insight into research findings (not competent).

These findings are in contrast with the anecdotal evidence obtained from the operational nurse managers who had indicated that some community service nurses may lack certain basic required competencies. This is especially true for the clinical patient care domain competencies which were rated the highest in terms of perceived competence, with more than 50.0% of respondents rating almost all clinical competencies as proficient. Clinical competency demands the acquisition of higher level behaviours in the cognitive, affective and psychomotor domains of learning and these competencies are required to achieve the delivery of high-quality nursing care (Khoza & Ehlers 2000). The findings of this study are also supported by other studies that found that community service nurses reported being confident in carrying out patient care activities (Govender et al. 2015). This was also confirmed by Hansen-Salie (2011) who found that majority of newly qualified professional nurses were perceived as somewhat competent in assessing health dimensions, developing nursing care plan for specific patients, identifying patients’ needs in care plans and in utilising technological advances to improve care and evaluating results of nursing care.

Of the clinical domain competencies, observations of vital signs, blood and urine testing, nebuliser therapy, oxygen therapy, checking and keeping record of medication appropriately, administration of medication according to the correct principles, undertaking the correct calculations to administer dosages and maintenance of fluid balance, had ratings of 60% and above by the respondents who rated community service nurses either proficient or advanced in these competencies. Two ethical and legal competencies (ensuring confidentiality and respect for patients, for example, right to privacy, were also rated as proficient or advanced by more than 60% of the respondents. These findings are also corroborated by Snell and Daniels (2014) who found that despite the difference between the competencies of a 4-year diploma programme and degree programme nurses as perceived by professional nurses, community service nurses were found competent in many dimensions irrespective of the difference in programmes they have completed before starting community service.

The lowest rated competence was for competencies related to quality assurance (8), five leadership competencies, three management competencies, three legal and ethical competencies and only one clinical competency (*developing accurate nursing care plans based on need*) (16.9% rated as not demonstrating competence). One of the key findings of the study was the perception that community service nurses did not demonstrate insight into latest research findings. Similar findings were also reported by Hansen-Salie (2011) who found that newly qualified nurses were perceived as not competent in critical thinking and research aptitude.

Leadership plays a central role in nursing practice and nursing leadership is identified as an essential component and competence-guiding nursing activity. The moderate findings of the study in the leadership competencies are supported by several researchers. Kelly and Courts (2007) reported that new professional nurse graduates were incompetent in leadership. This was confirmed by Wagensteen, Johansson and Nordstrom (2008) who stated that newly qualified professional nurses experienced the leadership role as challenging because of the lack of preparation with regard to clinical demands and also inability to prioritise the workload, planning and distribution of tasks. Further reasons for this were identified by López-Entrambasaguas et al. (2019) because of the fact that newly qualified professional nurses had not had the opportunity to develop leadership skills.

From the findings of this study, it can be identified that there are key areas in which the transition from a nursing student to a community service nurse should be further developed during the community service year. Brown and Crookes (2016) suggest that the newly graduated registered nurse is not ‘work ready’ and ‘does not feel ready’ and proper orientation programmes and development of the newly qualified registered nurse are essential. It would be important for institutions to ensure that autonomous practice and critical thinking are accompanied by the provision of adequate orientation and the availability of mentors and preceptors and can be supported through the use of information and communication technologies particularly in the rural areas (Abiodun et al. 2019). It would also be important for institutions to ensure the provision of adequate orientation and the availability of mentors and preceptors, and these processes can be supported through the use of information and communication technologies to facilitate autonomous practice and critical thinking (Abiodun et al. 2019). This is further corroborated by Makua (2016) who found that there is lack of professional development and support for community service nurses in most public institutions and that this may contribute to the lack of competency of these nurses. Makua (2016) therefore recommended that institutions must implement induction and professional development support programmes for newly qualified professional nurses during community service.

Several studies conducted by Benner (2001) cited in López-Entrambasaguas et al. (2019) studies revealed that the acquisition and development of competences occur along a continuum made up of five levels. This starts with being a novice, passing through an advanced beginner, competent and proficient before finally reaching the fifth stage of advanced competence. This process should be supported as shown by the work of Netshisaulu and Maputle (2018) who showed that as students lack confidence and skills to take up the new roles and accountability, role transition support and a foundation period of preceptorship for midwifery graduates at the start of their careers help midwives to make the journey from a novice to an expert (Netshisaulu & Maputle 2018). The authors also advocated that a contextual transition programme should be developed by institutions to orientate and mentor graduate midwives in their new roles before they could function as independent practitioners (Netshisaulu & Maputle 2018).

Lastly, there is a need to design the integration of competencies such as leadership and quality management into nursing curricula through teaching strategies that integrate leadership competences (Morrow 2015). This is supported by Roziers et al. (2014), who indicated that novice community service nurses require a structured orientation program and guided immersion in realistic clinical scenarios during their training in preparation for role transition. Mowry and Crump cited in Roziers et al.’s (2014) studies suggest that role-play of realistic clinical scenarios and immersion scenarios for role transition are effective for adult learners and build on simulation by incorporating realistic patient care environments and training in conflict management, assertiveness and practical ethics to foster realistic expectations and competence in dealing with ethical dilemmas. These strategies empower students with critical thinking skills in preparing them for the role of newly qualified nurses.

## Conclusion

The survey showed that the operational nurse managers perceived community service nurses as developing and proficient in most competencies and specifically so in the clinical patient care competencies. Newly qualified nurses need continuous development programmes during the period of CSN to build and sustain their confidence and skills with specific reference to non-direct care competencies of quality assurance and leadership. It can be interpreted that the completed program addressed the necessary competencies to be acquired by a professional nurse.

## Recommendations

### Implications for nursing education

- Assigning of preceptors to final-year students for the duration of the year to assist them to prepare for the role change to community service nurse (Roziers et al. 2014).
- The literature suggests that nursing education institutions should develop curricula that are more practice-based and which would help nursing students build enough skills and confidence prior to the commencement of community service period (Abiodun et al. 2019).

### Implications for nursing practice

- Supportive strategies for community service nurses should be developed such as orientation programmes that provide relevant information focussing on ensuring competencies in the clinical areas.
- Opportunities should be provided for community service nurses to become involved in activities that would increase their abilities and confidence. There is a wide concurrence that newly graduated nurses require a period of structured support following their graduation. A number of studies outside South Africa suggest that high-quality programmes are required to provide reliable support structures for newly graduated nurses in the first year of practice (Whitehead et al. 2013 cited in Abiodun et al. 2019 studies). The Nursing and Midwifery Council of United Kingdom recommends the development of globally accepted programmes of transition for a minimum period of 4 months to support newly graduated nurses (McCarthy & Murphy 2010 cited in Abiodun et al. 2019).
- The institution should maintain realistic expectations of community service nurses competency after completion of their training and assist them in competence development by continuous education programmes.
- Lastly, Rush et al. (2013) cited in Abiodun et al. studies (2019) recommend the development of public guidelines and clear policies that describe the expectations and responsibilities of newly graduated nurses in the transition programme. These would include guidelines directed at community service policy-makers, guidelines for supervision, guidelines for support and guidelines to clarify the role of community service nurses.

### Implications for nursing research

- Further research should be conducted in exploring the concept of a competency inventory for community service nurses with authentic assessment of competency by community services nurses themselves.

## Limitations

The individual competencies of community services nurses were measured by the perceptions of operational nurse managers and not by community nurses themselves. Competence was also not objectively measured.
